# Temporo-Spatial Dynamics of Event-Related EEG Beta Activity during the Initial Contingent Negative Variation

**DOI:** 10.1371/journal.pone.0012514

**Published:** 2010-09-02

**Authors:** Thomas Fischer, Robert Langner, Kersten Diers, Burkhard Brocke, Niels Birbaumer

**Affiliations:** 1 Chair of Differential and Personality Psychology, Department of Psychology, Technical University Dresden, Dresden, Germany; 2 Department of Psychiatry and Psychotherapy, RWTH Aachen University, Aachen, Germany; 3 Neuropsychology Section, Department of Neurology, RWTH Aachen University, Aachen, Germany; 4 Institute of Neuroscience and Medicine (INM-2), Research Center Juelich, Juelich, Germany; 5 Institute of Medical Psychology and Behavioral Neurobiology, Eberhard-Karls-University, Tübingen, Germany; 6 Istituto di Ricovero e Cura a Carattere Scientifico, Ospedale San Camillo, Venice, Italy; Tel Aviv University, Israel

## Abstract

In the electroencephalogram (EEG), early anticipatory processes are accompanied by a slow negative potential, the initial contingent negative variation (iCNV), occurring between 500 and 1500 ms after cue onset over prefrontal cortical regions in tasks with cue-target intervals of about 3 s or longer. However, the temporal sequence of the distributed cortical activity contributing to iCNV generation remains unclear. During iCNV generation, selectively enhanced low-beta activity has been reported. Here we studied the temporal order of activation foci in cortical regions assumed to underlie iCNV generation using source reconstruction of low-beta (13–18 Hz) activity. During the iCNV, elicited by a cued simple reaction-time task, low-beta power peaked first (750 ms after cue onset) in anterior frontal and limbic regions and last (140 ms later) in posterior areas. This activity occurred 3300 ms before target onset and provides evidence for the temporally ordered involvement of both cognitive-control and motor-preparation processes already at early stages during the preparation for speeded action.

## Introduction

The anticipation of a response signal after presenting a cue evokes a contingent negative variation (CNV) in the human electroencephalogram (EEG)[1]. The CNV starts around 500 ms after cue onset and lasts until the anticipated target occurs. During cue–target intervals of about 3 s or longer, the CNV consists of two temporally segregated components developing over different locations: an early component at anterior regions occurring 450–1500 ms after cue onset and a late component slowly rising up at premotor and posterior regions until the target appears [2], [3]. The first component is termed initial contingent negative variation (iCNV), the second is termed terminal contingent variation (tCNV) [4]. Much research has been done to explore the functional meaning of the two CNV components [see for instance 4,5]. The early component is thought to reflect an orienting response to the cue, whereas the late component is thought to express sustained-attention and motor-preparation processes [6]. In a study combining EEG and functional magnetic resonance imaging (fMRI), Nagai et al. [7] showed that the anterior cingulate cortex (ACC), the prefrontal cortex (PFC), and the supplementary motor area (SMA) are active during iCNV generation. Hamano and collaborators [8] found with subdural electrodes a patchy distribution of the cortical generators of the iCNV in prefrontal and supplementary motor areas. Lütcke et al. [9] found with fMRI for the early anticipation interval (i.e. during the typical period of iCNV generation) increased brain activity in motor and premotor cortical areas as well as the caudate nucleus.

In addition to these heterogeneous findings, several studies have investigated the temporo-spatial pattern of brain activity underlying the CNV globally, that is, without differentiating early and late components [3], [10], [11], [12]. To our knowledge, however, the temporo-spatial pattern of brain activity during the *early* CNV (iCNV) component has not been clearly shown yet.

During the iCNV, selectively enhanced oscillatory beta power was found in the EEG [13], [14], [15]. Molnar et al. [16] reported that event-related beta power during the CNV interval was maximal over anterior sites and higher during the iCNV interval compared to the tCNV interval. Alegre et al. [17] found maximal event-related beta activity over frontal sites of the cortex during the CNV interval. Some evidence regarding the temporal order of brain activity during the iCNV is provided by a study by Tallon-Baudry et al. [18], which reported temporal differences between event-related beta activities over different locations in a visual delayed matching-to-sample task. Maximal event-related beta activity was found at the frontal electrode about 700 ms after S1 onset and at the parietal one about 900 ms after S1 onset.

EEG beta rhythms have been found to represent activity in pyramidal cells subserving information transfer between distant brain structures [19], [20]. Therefore, beta activity during the iCNV may be a real-time reflection of information transfer of pyramidal cells necessary for preparatory control processes in anticipation of the target.

The main goal of this study was the identification of areas engaged in preparatory processes during the typical iCNV interval and the temporo-spatial pattern of their activity as revealed by event-related beta oscillations. The activity pattern was examined by analyzing the dynamics of the cortical projections of the time-varying cross-spectral density of the low-beta band.

## Methods

### Ethics statement

The study was approved by the local ethics committee of the Hospital of the University of Tuebingen. All subjects gave written informed consent prior to entering the study.

### Experiment

Twenty-eight healthy subjects (6 men, 18–33 years) participated in a forewarned reaction-time task. The paradigm consisted of an auditory cue (S1; 70 db, 1000 Hz, 200 ms), followed 4200 ms later by a target tone (S2; 70 db, 1500 Hz, 200 ms); the intertrial interval was 12 s. Stimuli were presented via in-ear phones. Subjects were instructed to keep their eyes closed, to hold their right hand on a computer mouse placed on the table in front of them, and to respond as quickly as possible to S2 with a click on the left mouse button. The data reported here resulted from one of three conditions of an experiment. The total duration of one condition was 20 min.

The EEG was recorded at 56 positions of the 10/10 system. Activity at both mastoids was recorded for optional re-referencing; two ground electrodes were placed at the earlobes. The vertical and horizontal eye movements were recorded with separate electrodes and were used later in addition to the 10/10 EEG electrodes for blind source separation with the second-order blind identification algorithm (SOBI) [21]. The EEG was amplified and recorded with a full-band DC-EEG system (neuroConn GmbH, Ilmenau, Germany) with a sampling rate of 512 Hz. After recording, a high-pass filter at 0.001 Hz and a low-pass filter at 150 Hz were used. The continuous EEG was segmented into 9000-ms epochs (starting 4000 ms before S1). Epochs with a high ratio of artifacts were rejected manually after visual inspection. After that the EEGLAB toolbox [22] was used to detect and correct artifacts (eye-blinks, muscle activity) after SOBI computation and for the computation of mean event related changes in the power spectrum, the event related spectral perturbations (ERSP) [23].

The values of the perturbations were averaged for frequencies between 13–18 Hz and for the time window starting 480 ms and ending 1660 ms after S1 onset. The averaged values were tested against the averaged value of a baseline interval (1502-314 ms before S1) for each channel.

### Source analysis

The exact low resolution brain electromagnetic tomography (eLORETA) software (freely available academic software at http://www.uzh.ch/keyinst/loreta.htm) was used to compute the cortical three-dimensional distribution of current density. The eLORETA method is a discrete, three-dimensionally distributed, linear, weighted minimum norm inverse solution. The eLORETA method has no localization bias even in the presence of structured noise and therefore is an improvement over the previously developed tomographies LORETA [24] and the standardized version, sLORETA [25]. A more detailed description of the method together with the proof of its exact zero-error localization property can be found in Pascual-Marqui [26]. In eLORETA, the intracerebral volume is partitioned in 6239 voxels at 5 mm spatial resolution.

For the eLORETA analysis, the artifact-corrected EEG was down-sampled to 256 Hz and 1024 time frames were exported from EEG-Lab to ASCII format. The epochs started 500 ms before S1 onset and ended 3500 ms after it. Time-varying cross-spectra were calculated using a continuous Gaussian window of 62 time frames (242 ms width). Subsequently, current source density of low-beta (13–18 Hz) oscillations was estimated in eLORETA for 6239 cortical voxels as a function of time. The mean of the baseline interval (380-32 ms before S1) was statistically tested against the mean of the iCNV interval (500–1500 ms) with a paired t-test without additional normalization and logarithmization. A permutation method using 5000 randomizations established the critical value for a significance level of 1%. Clusters that showed t-scores above 5.3 were used for further analysis.

## Results

**Figure 1:**
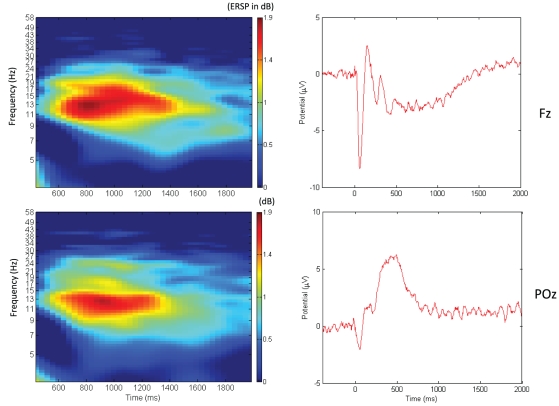
Event-related spectral perturbation (left panels) and event-related potential (right panels) at a frontal (upper panels) and a parietal (lower panels) electrode position. Time is relative to onset of warning signal (at zero milliseconds).

At the frontal electrodes, a negative wave occurred during the time interval of the iCNV, whereas a positive wave occurred at posterior electrode positions. Event-related spectral perturbations around the 13-Hz band were observed over anterior as well as posterior regions (see Figure 1).

**Figure 2:**
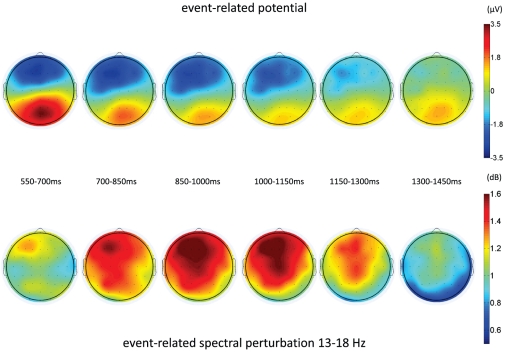
Topographies of the time course of the event-related potential and the event-related spectral perturbation.

The Bonferroni-corrected p-values of the paired t-tests showed significant perturbations in the iCNV interval for all EEG channels. The highest values for event-related spectral perturbations in the low-beta band were found in a left-central frontal cluster. This cluster included the electrodes AF3, AFz, F5, F3, F1, and Fz. The mean of this cluster (M = 1.118; SD = 1.106) was tested against the mean of a right-frontal cluster (AF4, F6, F2, FC4, FC6, C2; M = 0.999; SD = 0.958), a left-central posterior cluster (P5, P3, P1, Pz, PO3, POz; M = 0.863; SD = 0.943) and a right-posterior cluster (P6, P4, P2, T6, PO4, O2; M = 0.792; SD = 0.811). The Greenhouse–Geisser-corrected ANOVA for repeated measures showed a main effect of clusters [F (1.484, 40.074) = 5.649; p = .012; eta^2^ = .117]. Simple contrasts showed that beta power was significantly higher at the left-central frontal cluster compared to the right frontal [F(1, 27) = 5.644; p = .025; eta^2^ = .173], the left-central parietal [F(1, 27) = 6.001; p = .021; eta^2^ = .182], and the right parietal [F(1, 27) = 8.146; p = .008; eta^2^ = .232] clusters. As illustrated in Figures 1 and 2, the time course of peak values for event-related spectral perturbations and the evoked potentials was different. The maximal values of beta power occurred after the peak of the iCNV amplitude was reached.

### eLORETA

**Figure 3:**
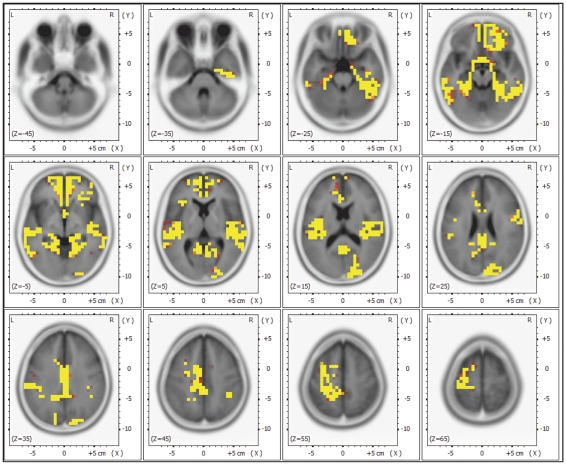
Regions of significant increases in event-related beta activity (t>5.3 compared to baseline).

The eLoreta statistic showed for the most part of the brain a significant increase of low-beta power during the iCNV interval (500–1500 ms after S1) compared to baseline (380-32 ms before S1). For further analysis, we specified clusters with a very high increase of event-related beta by choosing regions t>5.3 compared to baseline (see Figure 3). This way, 10 clusters were selected (see Table 1).

**Table 1:** Clusters of significant event-related beta activation.

*Brain Region*	*MNI-Coordinates*			*t-score*	*volume*	*Brodmann-Area*	*latency (SD) for 95%*
	*X*	*Y*	*Z*		*cm^3^*		*sec*
Left ACC	−7,	45,	0	5.88	4.3	10, 24, 32	0.748 (0.186)
Right inferior frontal gyrus	32,	32,	−15	5.43	5.0	11, 47	0.789 (0.220)
Left anterior insula	−32,	20,	5	5.51	2.1	13,45, 47	0.797 (0.262)
Subgenual ACC	0,	5,	−10	5.60	1.6	25	0.804 (0.192)
Left middle frontal gyrus	−25,	2,	45	6.10	1.4	6, 8, 32	0.834 (0.152)
Right temporal lobe	47,	−25,	5	5.99	3.5	13, 21, 22, 41	0.886 (0.264)
Left pre- and postcentral gyri	−35,	−27,	57	5.92	5.1	2, 3, 4, 6, 40	0.861 (0.195)
Left posterior insula	−35,	−30,	20	6.35	1.7	13, 41	0.828 (0.181)
Posterior cingulate gyrus	0,	−40,	30	5.93	5.5	23, 31	0.843 (0.213)
Right cuneus	20,	92,	20	5.85	2.6	18, 19, 31	0.873 (0.250)

For each cluster, the time-varying cross-spectral values were averaged. The time points of reaching 95% of the maximal value were determined for each cluster in a time window from 400 to 2000 ms after S1 onset.

**Figure 4:**
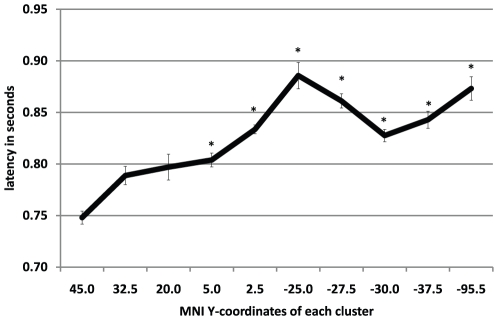
Means and standard errors of the time of reaching the threshold for each cluster, ordered in anterior to posterior direction. Asterisks indicate significant differences between the most anterior cluster and posterior ones.

The earliest activity (after 0.748 s; SD = 0.186) was found in the most frontal cluster, centered at y = 45 mm [anterior–posterior direction; coordinates refer to Montreal Neurological Institute (MNI) space] and including left middle frontal cortex and anterior cingulate gyrus. Simple contrasts showed that in this cluster, the 95% threshold was reached significantly earlier (p<.05) than in seven other, more posterior cortical clusters (all y<2.5, see Figure 4).

The temporo-spatial order of reaching the 95% threshold relatively earlier in anterior than posterior regions was further corroborated by the significant negative Spearman rank correlation (rho = −0.82, p<0.002) between time to threshold and MNI y-coordinate (anterior–posterior axis) of the 10 selected clusters.

## Discussion

During the iCNV interval, we found a broad distribution of significant event-related spectral perturbation for beta power during the interval of the early component of the CNV. Similar to Molnar et al. [16], the highest beta activity during the iCNV interval was found over anterior sites, especially in a left-central frontal cluster, which was congruent with the electrode sites that showed the typical negativity during the iCNV interval. The highest beta activation, however, was found after the iCNV peaked. Similar to the event-related-spectral perturbation, the cross-spectral power of the low-beta band increased significantly over most parts of the cortex. Regions with a very high increase in beta power were distributed all over the scalp.

A broadly distributed pattern of activity was also reported by previous studies using paradigms that evoked iCNVs and our regions overlapped with regions related to the CNV generation in previous fMRI studies [7], [9]. For instance, Lütcke et al. [9] found comparable blood oxygenation level-dependent (BOLD) responses in left and right insula as well as left motor cortex during the interval of CNV generation. In contrast to our results, Lütcke et al. [9] associated bilateral insula activity with the late component of the CNV. On the other hand, Lütcke et al. [9] associated the BOLD response in the left postcentral cortex with the early CNV, which agrees with our results of event-related beta responses in this region. Activity in left insula and left postcentral cortex during the interval of the early CNV was also reported by Nagai et al. [7].

The temporal order of this neural activity, however, could not be elucidated because of the insufficient time resolution of fMRI. Our major aim was to uncover the temporal order in the interplay between different parts of the network that are active 550 to 1450 ms after cue onset. Our results revealed a temporal order of cluster-specific maxima in beta amplitudes in anterior–posterior direction: First, beta power peaked significantly earlier in anterior than in posterior areas. Second, there was a significant correlation between clusters y-coordinates (anterior–posterior axis) and the onset latencies of the beta peaks in the different clusters. Therefore, brain activity during the iCNV-related time interval (500–1200 ms after cue onset) appears to be characterized by a specific temporo-spatial pattern, evolving from anterior to posterior regions.

The early activity in the left and mid-ACC provides additional evidence for the crucial role of the ACC for the cue-induced expectation of upcoming events. The specific role of the ACC during the iCNV time interval might, at least in part, lie in the motivationally guided mobilization of preparatory processes via the adjustment of cortical arousal in order to optimize upcoming information processing. The ACC may realize this mobilization via pyramidal cell connections with the amygdala and the locus coeruleus [27], [28] as well as with the anterior and midline thalamic nuclei, which are known to participate in regulating cortical arousal [29]. On the other hand, the ACC and anterior insula have been associated with the implementation and maintenance of task sets across a wide range of cognitive tasks [30]. This would agree with a viewing the iCNV, which has previously been associated with the orienting reaction, as reflecting an orienting process towards a memory representation of the task in order to reactivate the task goal. The right inferior frontal area, which also peaked early, has been found active during the processes of inhibition of habituated motor processes [31].

These findings suggest that these areas may be involved in activating the appropriate task set by modulating computational activity in more posterior areas. It is tempting to conjecture that the parallel early activation peaks in ACC, insula and PFC reflect the initiation of two parallel streams of control, a basal energetical and a higher-order computational one, that are both essential for optimal task performance. Coull et al. [32] found BOLD responses in similar regions, the right inferior frontal cortex and the left anterior insula, during the performance of a simple forewarned reaction-time task with stimulus asynchrony onsets between 750 and 1550 ms. The authors especially related activation in the inferior frontal regions to the reorienting of the attentional focus. Although we cannot explain the exact function of the increased event-related beta activity within the identified regions based on our data, we do think that we made an important further step towards delineating the different functional networks that subserve the preparation for speeded responses.

In conclusion, the increased low-beta activity may reflect activity in long axons of pyramidal bundles [19], interconnecting the cortical networks involved in implementing attentional, energetical and computational functions necessary for the preparation for action. The study demonstrated a specific temporo-spatial pattern of preparation-related subprocesses, expressed by the generation of enhanced beta power during the iCNV interval, with peak activity moving through time from anterior to posterior brain regions.
